# What can we learn from the features and presentation of retinal pigment epithelium hypertrophy?

**DOI:** 10.5935/0004-2749.2025-0048

**Published:** 2025-04-04

**Authors:** Gustavo Rosa Gameiro, Maura Abraham-Marin, Zelia Maria Correa

**Affiliations:** 1 Bascom Palmer Eye Institute, University of Miami Miller School of Medicine, Miami, FL, USA; 2 Sylvester Comprehensive Cancer Center, University of Miami Miller School of Medicine, Miami, FL, USA

Dear Editor,

We read with great interest the article by Carvalho et al., describing the use of congenital hypertrophy of the retinal pigment epithelium (CHRPE) as a phenoty-pic marker for familial adenomatous polyposis (FAP)^(1)^. The study highlights the importance of CHRPE as a risk factor for FAP, particularly in cases with bilateral fish-tail lesions^(2^,^3)^. This is an informative and important finding that motivated us to add further insights into the clinical spectrum of retinal pigment epithelium (RPE) hypertrophy through a review of the three distinct presentations. Knowledge of these is critical for accurate diagnosis and differentiation.

**Figure 1:**
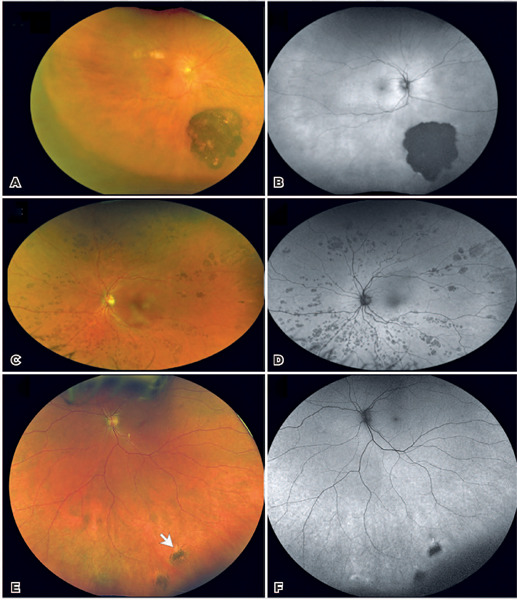
Clinical spectrum of retinal pigment epithelium (RPE) hypertrophy. Solitary RPE hypertrophy (A and B). A. A wide-field color fundus photograph showing a large flat dark gray to black lesion with scalloped margins in the inferotemporal periphery associated with lacunae skin pigmentation; **B**. Autofluorescence (AF) showing sharp lesion margins and blockage of the autofluorescence despite the areas of lost pigment; **Bear-track lesions (C and D). C**. A wide-field color fundus photograph showing diffuse small light-gray patches of retinal pigment epithelium hyperplasia in a clumping pattern consistent with the “bear-track” configuration; **D**. AF image in which lesions completely block the fundus fluorescence. ***Fish-tail lesions* (E and F). E**. A wide-field color fundus photograph showing two small gray-black flat lesions in the inferior periphery with associated whitish fish-tail edges (indicated by the white arrow); **F**. AF image in which the lesions block some of the AF showing. The hyper-AF associated with the fish-tail effect can be seen at the lesion margins.

1. ***Solitary RPE hypertrophy*** (Figures 1A and 1B): Although these are sometimes called CHRPE, they have not been definitively proven to be congenital. They appear as single, broad-base gray-black spots in fundus photography and are commonly located in the peripheral retina. They are characterized by sharp margins and a shadow effect that mimics thickness, making these an important differential diagnosis with choroidal melanoma. Some chronic lesions may present with patches of lost melanin pigment, leading to lacunae that can enlarge over time. These lesions are sporadic, asymptomatic, and predominantly located in the fundus periphery. They are not associated with systemic conditions. Rare instances have been reported of neoplasms such as melanoma and adenocarcinoma arising *de novo* from extensive RPE^(4^,^5)^.
2. ***Bear-track lesions*** (Figures 1C and 1D): Bear-track lesions are so named because they manifest as clusters of small pigmented lesions resembling bear paw prints. While congenital, bear tracks are sporadic and unrelated to FAP. Their lack of any specific systemic associations and the absence of fish-tail depigmentation distinguish them from FAP-associated CHRPE.
3. ***Fish-tail lesions*** (Figure 1E and 1F): These ovoid lesions exhibit a unique fish-tail-like area of depigmentation at one margin. They are most often associated with FAP. Their bilaterality and tendency to appear as multiple lesions (typically 2–6 per eye) make them a significant phenotypic marker for this condition. Recognizing these features is essential for early identification and screening of at-risk patients.

We believe a more detailed discussion of these forms of RPE hypertrophy would add to the utility of Carvalho et al.’s findings and assist your readers in distinguishing between FAP-associated lesions and other RPE abnormalities.^(2)^

We also feel that the use of the term “congenital” in this context is problematic as there is no evidence to indicate, which if any of these variations are truly congenital. The lesions are known to grow in more than 50% of cases. In clinics, patients with RPE hypertrophy merit an annual fundus evaluation to monitor for lesion enlargement and the other aforementioned risks.

We commend the authors for their significant contribution and for raising awareness of CHRPE as an important screening finding. We hope the points we have made will broaden the discussion of this condition and further emphasize the importance of early diagnosis and multidisciplinary approaches to hereditary syndromes like FAP.
